# Prevalence of drug-resistant tuberculosis in Nigeria: A systematic review and meta-analysis

**DOI:** 10.1371/journal.pone.0180996

**Published:** 2017-07-13

**Authors:** Cajetan C. Onyedum, Isaac Alobu, Kingsley Nnanna Ukwaja

**Affiliations:** 1 Department of Medicine, College of Medicine, University of Nigeria, Enugu Campus, Enugu, Nigeria; 2 National Tuberculosis and Leprosy Control Programme, Ministry of Health, Abakaliki, Ebonyi State, Nigeria; 3 Department of Internal Medicine, Federal Teaching Hospital, Abakaliki, Ebonyi State, Nigeria; Institut de Pharmacologie et de Biologie Structurale, FRANCE

## Abstract

**Background:**

Drug-resistant tuberculosis (TB) undermines control efforts and its burden is poorly understood in resource-limited settings. We performed a systematic review and meta-analysis to provide an up-to-date summary of the extent of drug-resistant TB in Nigeria.

**Methods:**

We searched PubMed, Scopus, Embase, HINARI, AJOL, the Cochrane library, Web of Science, and Google Scholar for reports published before January 31 2017, that included any resistance, mono-resistance or multidrug resistance to anti-TB drugs in Nigeria. Summary estimates were calculated using random effects models.

**Results:**

We identified 34 anti-TB drug resistance surveys with 8002 adult TB patients consisting of 2982 new and 5020 previously-treated cases. The prevalence rate of any drug resistance among new TB cases was 32.0% (95% CI 24.0–40.0%; 734/2892) and among previously-treated cases, the rate was 53.0% (95% CI 35.0–71.0%; 1467/5020). Furthermore, multidrug resistance among new and previously-treated cases was 6.0% (95% CI 4.0–8.0%;161/2502)and 32.0% (95%CI 20.0–44.0; 357/949), respectively. There was significant heterogeneity between the studies (p<0.001, I^2^ tests). The prevalence of drug-resistant TB varied according to methods of drug susceptibility testing and geographic region of Nigeria.

**Conclusion:**

The burden of drug-resistant TB in Nigeria is high. We recommend that a national anti-TB drug resistance survey be carried out, and strategies for case detection and programmatic management of drug-resistant TB in Nigeria need to be strengthened.

## Background

According to the World Health Organisation (WHO) global report 2016, among 10.4 million incident TB cases worldwide, 3.9% are estimated to have had rifampicin- or multidrug-resistant tuberculosis (MDR/RR-TB) in 2015 [1]. In addition, 21% of previously treated TB cases were estimated to have had MDR/RR-TB in the same year [1]. MDR-TB is caused by strains of *M*. *tuberculosis* that is resistant to both isoniazid and rifampicin. Drug-resistant TB (DR-TB) patients require prolonged and expensive treatment using second-line medications that are less effective and more toxic [1]. The acquisition or emergence *M*. *tuberculosis* resistance may occur from; previous exposures to quinolones [2], use of inferior regimens [3], poor adherence to anti-TB drug, previous TB treatment [4–5], and high human immunodeficiency virus (HIV) co-infection [6–7]. The diagnosis of drug-resistance require patients to be tested for susceptibility to anti-TB drugs, either by conventional (phenotypic) drug susceptibility testing (DST) or rapid molecular diagnostic (genotypic) methods [1]. The WHO recommends all presumptive TB patients to undergo DST although many countries still lack laboratory capacity to achieve this [1]. Therefore, in most low- and middle-income countries, there may be a high level of under-diagnosis and misdiagnosis of DR-TB. However, with the recent recommendation of rapid molecular diagnostic tests as a first-line TB screening test [1], there is progressive increase in reporting of DR-TB in resource-limited settings [8–9].

Nigeria is one of the countries included among the 30 high burden countries for TB, TB/HIV and DR-TB [1]. According to the WHO, the estimated incidence of TB in Nigeria is 322 per 100 000 population with only 15% of the total burden of the disease in the country being notified in 2015 [1]. The WHO estimates that the proportion of patients with MDR/RR-TB is 4.3% among new cases and 25% among previously-treated cases in Nigeria [1]. With the increasing utilisation of the newer molecular diagnostic techniques for TB diagnosis and the current advocacy for a country-wide roll-out by the Nigeria National TB Programme and other development partners [1, 10], several studies have reported on the rates of DR-TB in different cohorts of TB patients across various settings in Nigeria. However, as most of these studies were based on small sample local or facility-based data, a comprehensive analysis of the burden of DR-TB from different parts of Nigeria is urgently needed. Quantification of a reliable estimate of the extent of DR-TB is crucial to guide intervention policies for programmatic management strategies and for antimicrobial resistance monitoring. We therefore conducted a systematic review and meta-analysis of published data to provide a comprehensive and up-to-date assessment of the burden of DR-TB in Nigeria.

## Methods

### Data sources and search strategy

We searched the following databases: PubMed/MEDLINE, HINARI, Embase, AJOL, the Cochrane library, Web of Science, Google Scholar (top 800 papers), and Scopus for studies published before January 31 2017, which reported on the prevalence or incidence of DR-TB in Nigeria among patients with new or previously treated TB. Keywords from Medical Subject Headings or titles or abstracts of the studies were searched for with the help of Boolean operators (and, or) without language limitations. Search terms used included “tuberculosis” or “Mycobacterium tuberculosis” and “drug resistance” or “drug susceptibility”, anti-TB resistance, DR-TB, MDR/RR-TB, (isoniazid or rifampicin or ethambutol or streptomycin) resistant TB, and Nigeria. Details of the full search strategy for one of the databases are as shown in S1 Table. Additionally, we reviewed the reference lists of primary studies and review articles.

### Inclusion and exclusion criteria

All studies in which prevalence of DR-TB were reported in a given period were included. Also, the included original articles must report on some or all of the following: the prevalence of any resistance, mono-resistance or multidrug resistance to anti-TB drugs. Only studies containing data regarding the proportion of DR-TB among new cases or previously-treated cases were included. Also, included were studies that referenced a standard method for phenotypic or genotypic DST for *M*. *tuberculosis* against first-line anti-TB drugs (isoniazid, rifampicin, ethambutol, streptomycin). Studies with the following characteristics were excluded from the analysis: studies on non-tuberculous mycobacterium, articles on extrapulmonary TB or childhood TB; studies reporting on prevalence of an undefined mixture of new and previously-treated patients, and studies not evaluating DST based on first-line anti-TB drugs. Editorials, narrative review articles, case reports, and conference abstracts, as well as duplicate publications were excluded from the analysis.

### Data extraction and quality assessments

Two reviewers (KNU and AI) independently screened each title and abstract and resolved discrepancies by consensus. We obtained full texts of citations selected for review, and the reviewers extracted all study data independently, resolving discrepancies by consensus. For all studies, the following data were extracted: first author, year of publication, study setting, study enrolment time, DST method, the number of patients investigated, and drug resistance status. KNU and AI independently assessed the quality of the studies using the Joanna Briggs Institute’s critical appraisal checklist for assessment of quality for studies reporting prevalence data [11].

### Operational definitions

We used standard definitions as previously described [1, 8–9]. DR-TB among new TB cases refers to resistance among patients who have never received first-line anti-TB drugs. DR-TB among previously-treated TB cases refers resistance among patients who had previously received first-line anti-TB drugs. Mono-resistance was defined as resistance to only one first-line anti-TB drug. MDR-TB was defined as resistance to at least isoniazid and rifampicin. Any drug resistance was defined as resistance to one or more first-line TB drugs regardless of mono-resistance or MDR. Genotypic DST method is defined as a DST technique which utilised a WHO-certified nucleic acid amplification technology (molecular method) to diagnose the drug-resistant TB i.e., Xpert^®^ MTB/RIF and molecular line probe assays. Phenotypic DST method is defined as a DST technique which utilised a WHO-recognised conventional testing method to diagnose the drug-resistant TB i.e., studies which utilised a liquid culture system, or any of the three solid culture methods (proportion method, resistance ratio method, or absolute concentration method) [1,12].

### Statistical analysis

The pooled prevalence and 95% confidence intervals (95% CIs) were calculated using random effects model based on the exact binomial method of Hamza et al fitted using *metaprop*in STATA 13.1 (Stata Corp, College Station, Texas, USA) [13–14]. The χ^2^ based Q statistic and I^2^ test were used to assess the between-study heterogeneity using two-sided P-values. Begg rank correlation test and funnel plot was used to assess publication bias using the Comprehensive Meta-Analysis software 2.2 (Biostat Inc, USA) [15–16].

**Fig 1:**
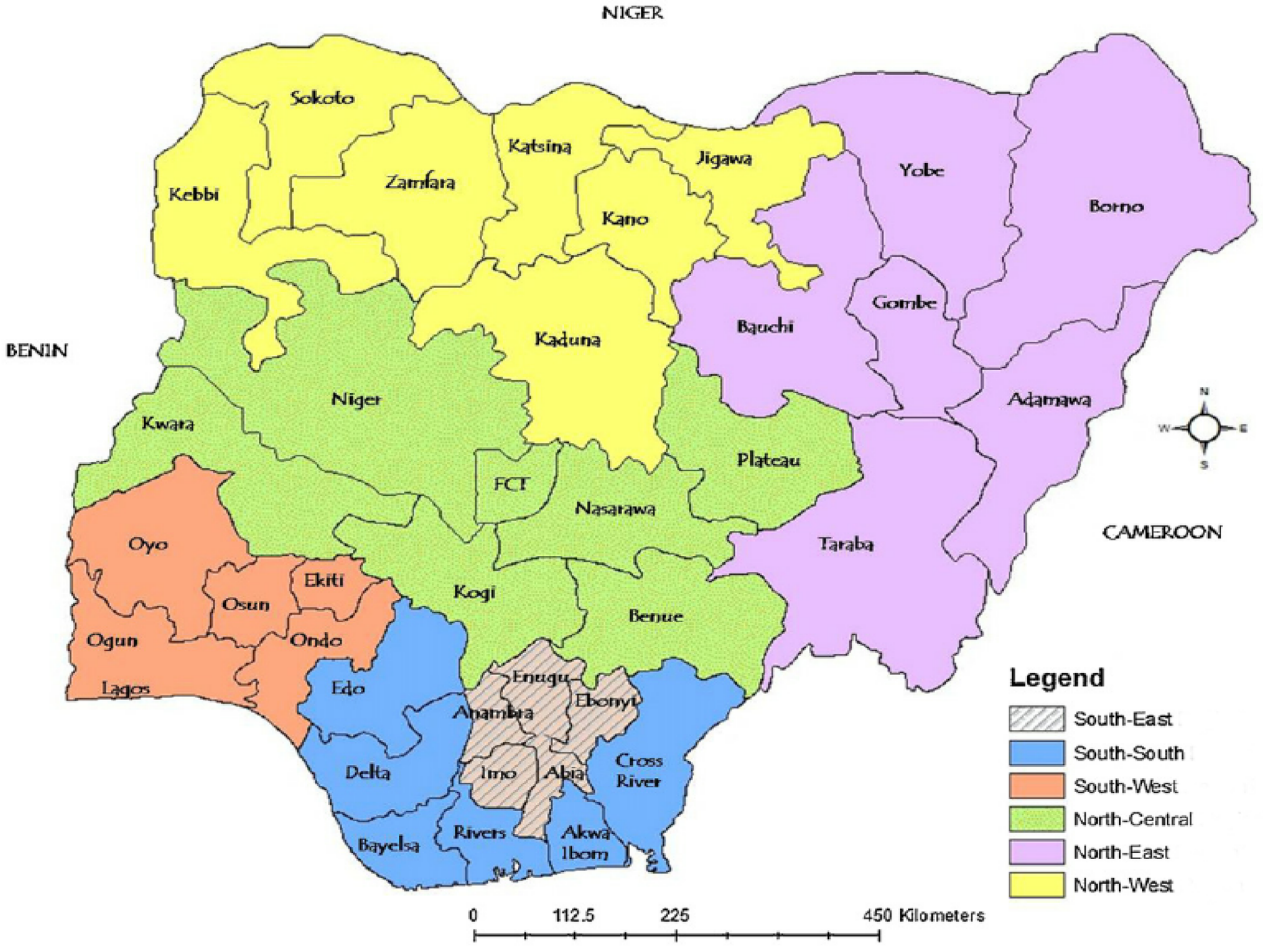
Map of Nigeria indicating the geopolitical zones of the country. [Northern region: North-west, North-central, North-east; Southern region: South-west, South-east, South-south].

Also, Nigeria is divided into two regions (north and south) with this further subdivided into six geopolitical zones (north-west, north-central, north-east, south-west, south-east and south-south) shown in Fig 1. We carried-out sub-group analyses of the DR-TB prevalence rate based on DST (phenotypic or genotypic) method and geographic region (northern versus southern Nigeria).

## Results

### Characteristics of studies

**Fig 2:**
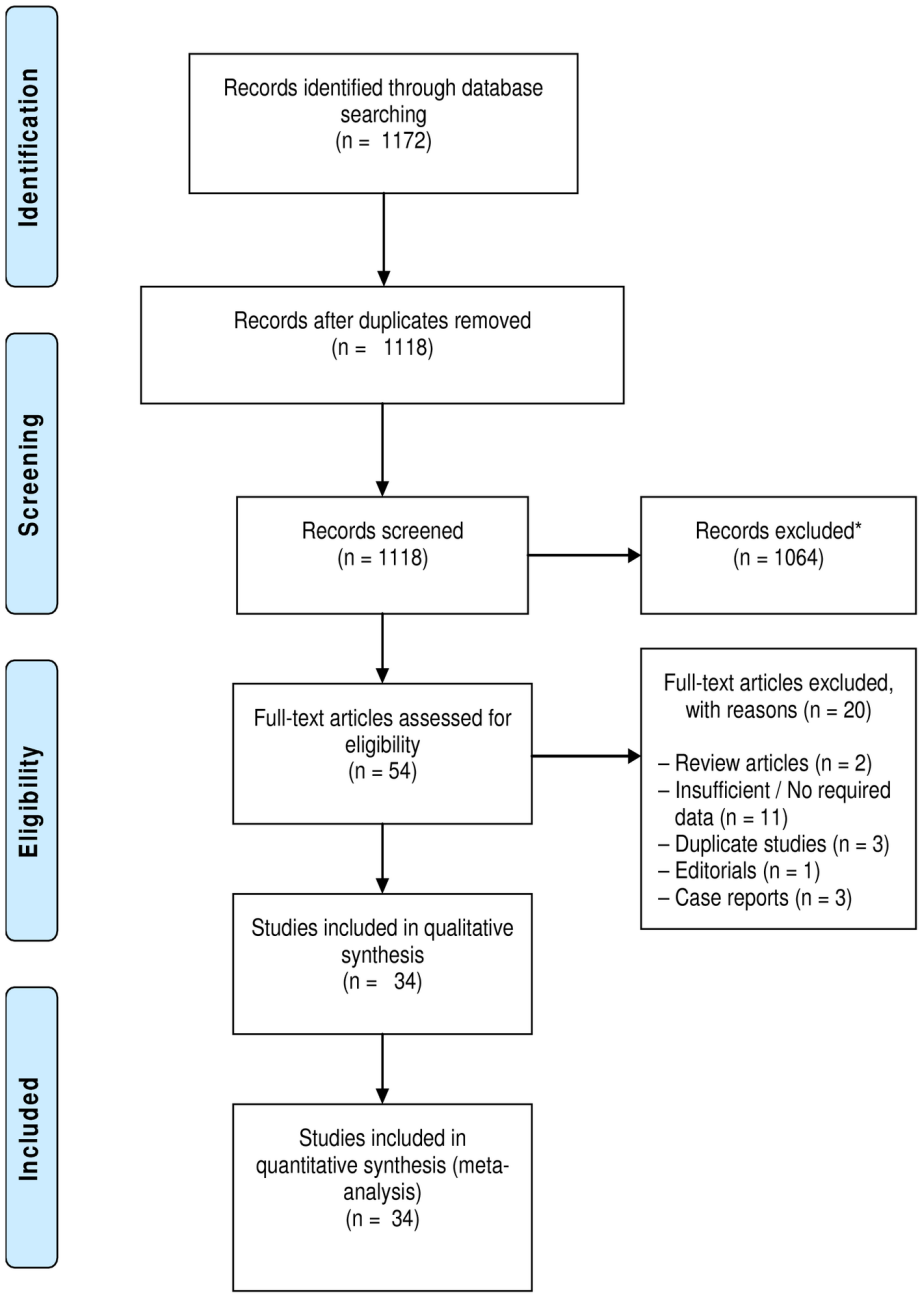
Flow chart depicting the study selection process. (*Records excluded due to lack of relevance).

A total of 1172 articles were retrieved by literature search Fig 2. Of these, 1118 articles were retained after duplicates were removed; 1064 of them were excluded due to irrelevance based on their title and abstract, and 54 were retained for full-text evaluation. Finally, after a detailed full-text evaluation, 34 articles published between 1975 and 2016 were included [17–50]. The distribution of the studies and relevant data retrieved for this analysis are summarised in Table 1. Most of the studies were conducted in south-west zone of Nigeria (n = 12) compared with north-central (n = 8), north–west (n = 6), south-east (n = 3), south-south (n = 2) and north-east (n = 1). Overall, 17 studies were from the southern region, 15 were from the northern region and 2 studies were conducted across multiple zones of the country. Regarding DST methods, 23 studies used the phenotypic methods (proportion, absolute concentration and the BACTEC system) and 11 studies used genotypic methods (Xpert MTB/RIF and Genotype MTBDR*plus* line-probe assays). Of the 34 articles, 11 provided data on new TB cases only, another 11 provided data on retreatment cases only, and 12 provided data on both new and retreatment cases, Table 1. A total of 8002 TB patients consisting of 2982 new and 5020 previously-treated TB cases were analysed.

**Table 1:** Included studies after full-text evaluation.

First author	Published year	Enrolment time	Geographic zone	Diagnostic method	Total	New cases				Previously-treated cases			
						Case number	Any	Mono	MDR	Case number	Any	Mono	MDR
Dosunmu	2008	2004–2005	North-central	Phenotypic	500	500	20	-	20	-	-	-	-
Lawson	2010	2007	North-central	Phenotypic	32	-	-	-	-	32	14	10	4
Lawson	2011	2009–2010	MZ	Phenotypic	426	357	259	20	18	69	69	-	13
Uzoewulu	2014	2009–2011	South-east	Phenotypic	180	151	56	23	6	29	27	11	8
Nwachukwu	2016	2013–2014	South-east	Genotypic	389	389	12	-	-	-	-	-	-
Pokam	2013	2008–2009	South-south	Phenotypic	58	58	35	13	6	-	-	-	-
Otu	2013	2011–2012	South-south	Phenotypic	100	100	42	17	4	-	-	-	-
Aghaji	2010	2003–2005	South-east	Phenotypic	82	-	-	-	-	82	81	1	59
Gidado	2015	2011–2013	MZ	Genotypic	3669	-	-	-	-	3669	815	-	-
Halilu	2013	2011–2012	North-east	Genotypic	300	-	-	-	-	300	22	-	-
Aliyu	2013	2010–2011	North-west	Genotypic	375	324	15	12	3	51	8	6	2
Fawcett	1975	1973	North-west	Phenotypic	61	61	7	6	-	-	-	-	-
Rikoto	2015	2013–2014	North-west	Phenotypic	81	38	15	5	10	43	43	11	32
Kolo	1991	1987–1989	North-west	Phenotypic	86	75	41	5	0	11	8	5	1
Adamu	2015	2013–2014	North-west	Genotypic	339	298	11	2	9	41	32	5	27
Rasaki	2015	2013	North-west	Genotypic	54	-	-	-	-	54	10	-	-
Nwofor	2015	2012–2013	North-central	Phenotypic	97	-	-	-	-	97	14	8	6
Idigbe	1992	1987–1990	South-west	Phenotypic	96	-	-	-	-	96	54	33	-
Egbe	2016	2015	North-central	Genotypic	91	91	6	-	-	-	-	-	-
Daniel	2011	2007–2009	South-west	Phenotypic	67	23	3	3	0	44	38	4	34
Oluwaseun	2013	2011	South-west	Phenotypic	69	69	24	9	9	-	-	-	-
Okodua	2012	2008–2010	South-west	Phenotypic	103	103	45	17	18	-	-	-	-
Bello	2014	2013 -	South-west	Genotypic	48	-	-	-	-	48	9	-	-
Kehinde	2012	2011	South-west	Genotypic	24	-	-	-	-	24	5	3	1
Kehinde	2013	2011	South-west	Genotypic	6	6	3	1	2	-	-	-	-
Kehinde	2007	2005–2006	South-west	Phenotypic	56	56	30	0	30	-	-	-	-
Gehre	2016	2009–2013	South-west	Phenotypic	173	41	19	8	9	132	96	15	75
Olusoji	2011	2007–2009	South-west	Phenotypic	88	23	4	2	0	65	51	5	42
Eltayeb	2011	2007–2010	South-west	Phenotypic	82	-	-	-	-	82	52	-	46
Sogaolu	2012	2008–2011	South-west	Phenotypic	82	68	23	10	4	14	3	2	0
Ani	2009	2006–2007	North-central	Phenotypic	61	50	12	9	2	11	6	3	2
Mawak	2006	1997–2000	North-central	Phenotypic	35	35	12	8	0	-	-	-	-
Ukaegbu	2016	2013	North-central	Genotypic	83	66	40	29	11	17	6	5	1
Ukoli	2012	2008–2010	North-central	Phenotypic	9	-	-	-	-	9	-	-	4

MZ = multiple zones; (–) indicates not applicable Any = any resistance; Mono = mono-resistance; MDR = multidrug resistance

### Quality assessment

Across the 10 quality domains evaluated, majority of the studies met five or more of the quality criteria. Most of the studies (n = 19) met 7 to 10 of the quality criteria assessed, and others (n = 15) met 5 to 6 of the quality criteria assessed for prevalence studies, S2 Table. The most common quality criteria failed by the studies were: inadequate sample size, poor statistical analytical strategy, not evaluating for confounders and non-reporting of results for sub-groups S2 Table.

### Anti-tuberculosis resistance among new TB patients

**Fig 3:**
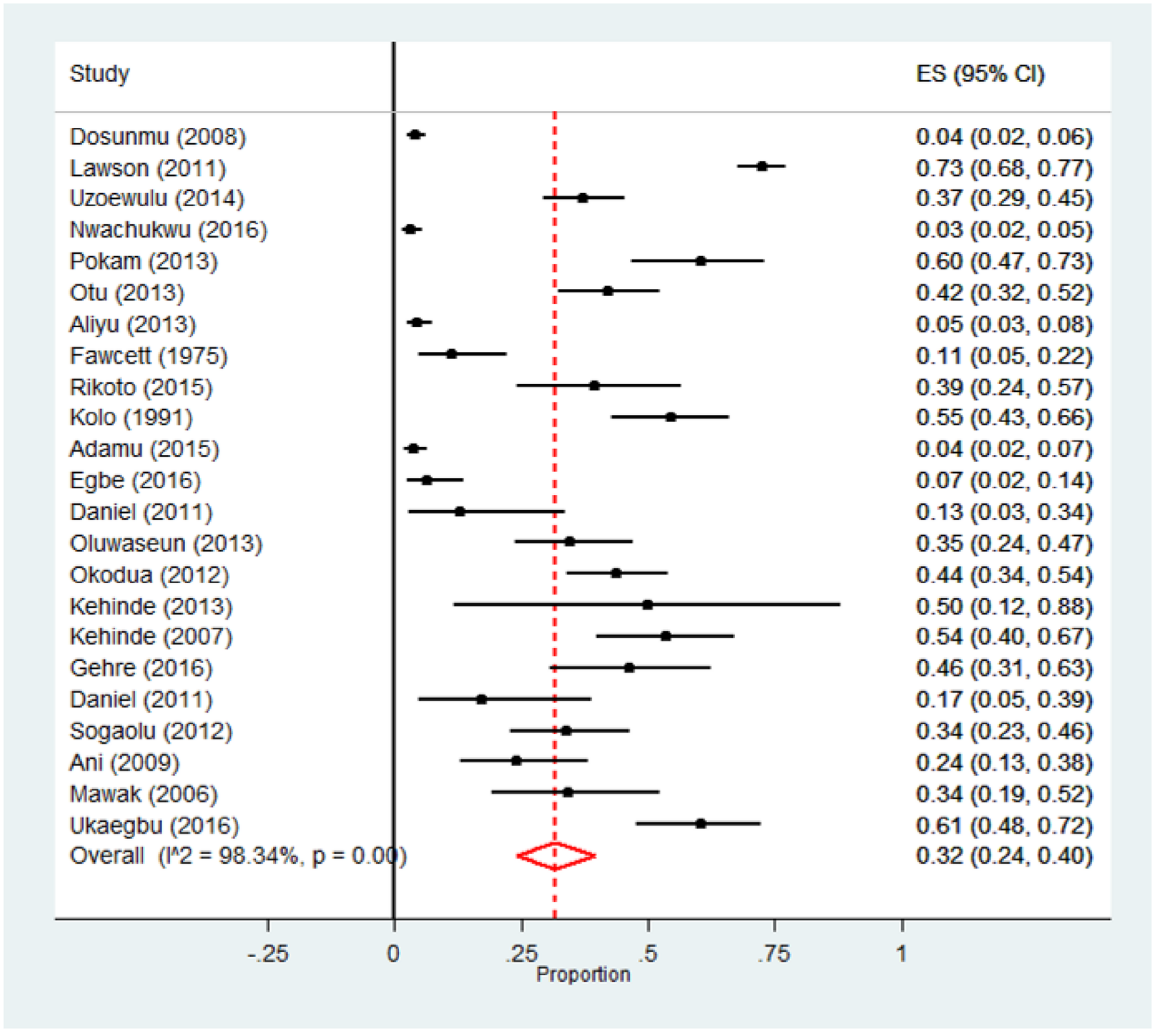
Forest plot of the meta-analysis on any drug resistance in new TB cases. [CI: confidence interval].

**Fig 4:**
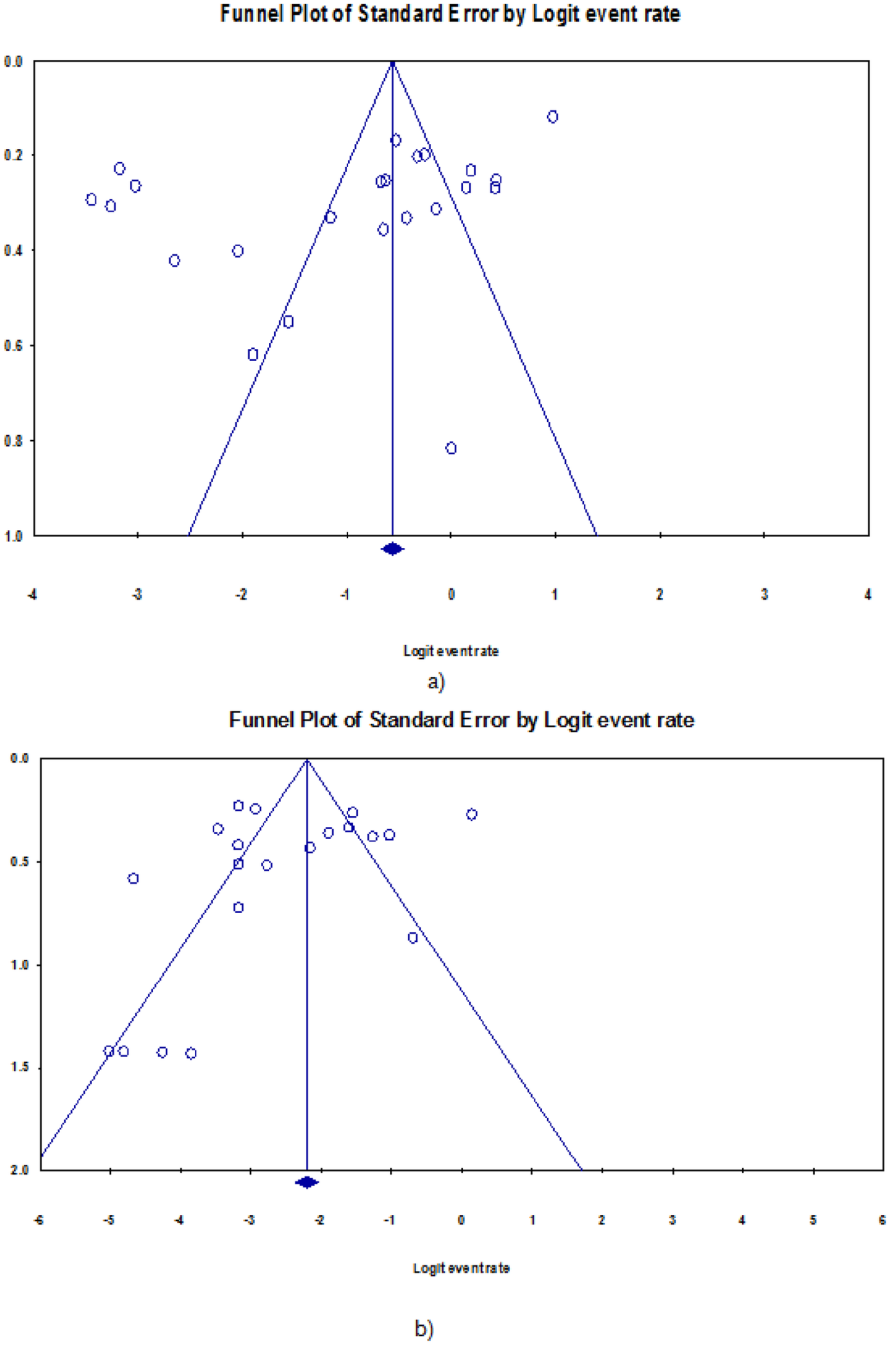
Funnel plot of the meta-analysis on prevalence of: a) any drug resistance, b) multidrug resistance among newly-diagnosed TB patients, Nigeria.

Table 2 presents the pooled analysis of the burden of DR-TB among newly diagnosed TB patients in Nigeria. The prevalence of any drug resistance among new cases was 32.0% (95% CI 24.0–40.0%; 734/2892). However, evident heterogeneity was observed (P <0 .001). Fig 3 shows the forest plot of the meta-analysis of any anti-TB resistance in new TB cases. Also, as shown in Fig 4, little evidence for publication bias was observed (Begg rank correlation analysis P = 0.101). There was a wide variation in the rate of any resistance among new TB cases between studies utilising phenotypic and genotypic methods of DST (37.0 vs. 12.0%) respectively; and the difference was significant (χ^2^ = 8.56, P < 0.001). In addition, pooled rates for the northern region (21.0%) were lower compared to the southern Nigeria (36.0%); but the between group difference was not significant (χ^2^ = 3.08, P = 0.08). The rate of anti-TB mono-resistance among new patients was 13.0% (95% CI 10.0–17.0%; 199/2002); this rate varied with region and DST methods, Table 2. Overall, the pooled prevalence of MDR-TB among new TB patients in Nigeria was 6.0% (95% CI 4.0–8.0%;161/2502), there was little variation according to DST method—occurring in 4.0% and 7.0% of patients diagnosed using genotypic and phenotypic methods, respectively; (χ^2^ = 1.45, P = 0.23). Also, we observed little evidence for publication bias (Begg rank correlation analysis P = 0.601) and Fig 4. The pooled prevalence of MDR-TB was 3.0% (95% CI 1.0–5.0; 55/1447) in northern and 12.0% (95% CI, 7.0–18.0; 88/698) in southern Nigeria; (χ^2^ = 9.59, P < 0.001). S1 Fig shows the forest plot of the meta-analysis of MDR-TB in new TB cases in Nigeria.

**Table 2:** Pooled event rates of drug resistant tuberculosis among newly-diagnosed TB patients, Nigeria.

Variables	Sub-group	Pooled event rate % (95% CI)	n/N	No. of Studies	Heterogeneity I^2^ (P -value)
**Any drug resistance**	Total	32.0 (24.0–40.0)	734/2892	23	98.3 (<0.001)
	Stratified by DST				
	Genotypic	12.0 (6.0–18.0)	87/1174	6	94.8 (<0.001)
	Phenotypic	37.0 (22.0–52.0)	647/1808	17	98.4 (<0.001)
	Stratified by region^a^				
	North	21.0 (15.0–28.0)	179/1538	10	95.6 (<0.001)
	South	36.0 (21.0–51.0)	296/1087	12	97.0 (<0.001)
**Mono-drug resistance**	Total	13.0 (10.0–17.0)	199/2002	20	89.9 (<0.001)
	Stratified by DST				
	Genotypic	11.0 (4.0–18.0)	44/694	4	94.7 (<0.001)
	Phenotypic	13.0 (10.0–17.0)	155/1308	16	64.6 (0.001)
	Stratified by region^a^				
	North	12.0 (0.07–0.17)	76/947	8	90.0 (<0.001)
	South	16.0 (13.0–18.0)	103/698	11	0 (0.90)
**Multidrug resistance**	Total	6.0 (4.0–8.0)	161/2502	21	86.1 (<0.001)
	Stratified by DST				
	Genotypic	4.0 (0.0–8.0)	25/694	4	82.5 (<0.001)
	Phenotypic	7.0 (4.0–10.0)	136/1808	17	86.2 (<0.001)
	Stratified by region^a^				
	North	3.0 (1.0–5.0)	55/1447	9	77.5 (<0.001)
	South	12.0 (7.0–18.0)	88/698	11	87.2 (<0.001)

DST = drug susceptibility testing method; TB = tuberculosis;

^a^ = studies from multiple regions excluded

### Anti-tuberculosis resistance among previously-treated TB patients

**Fig 5:**
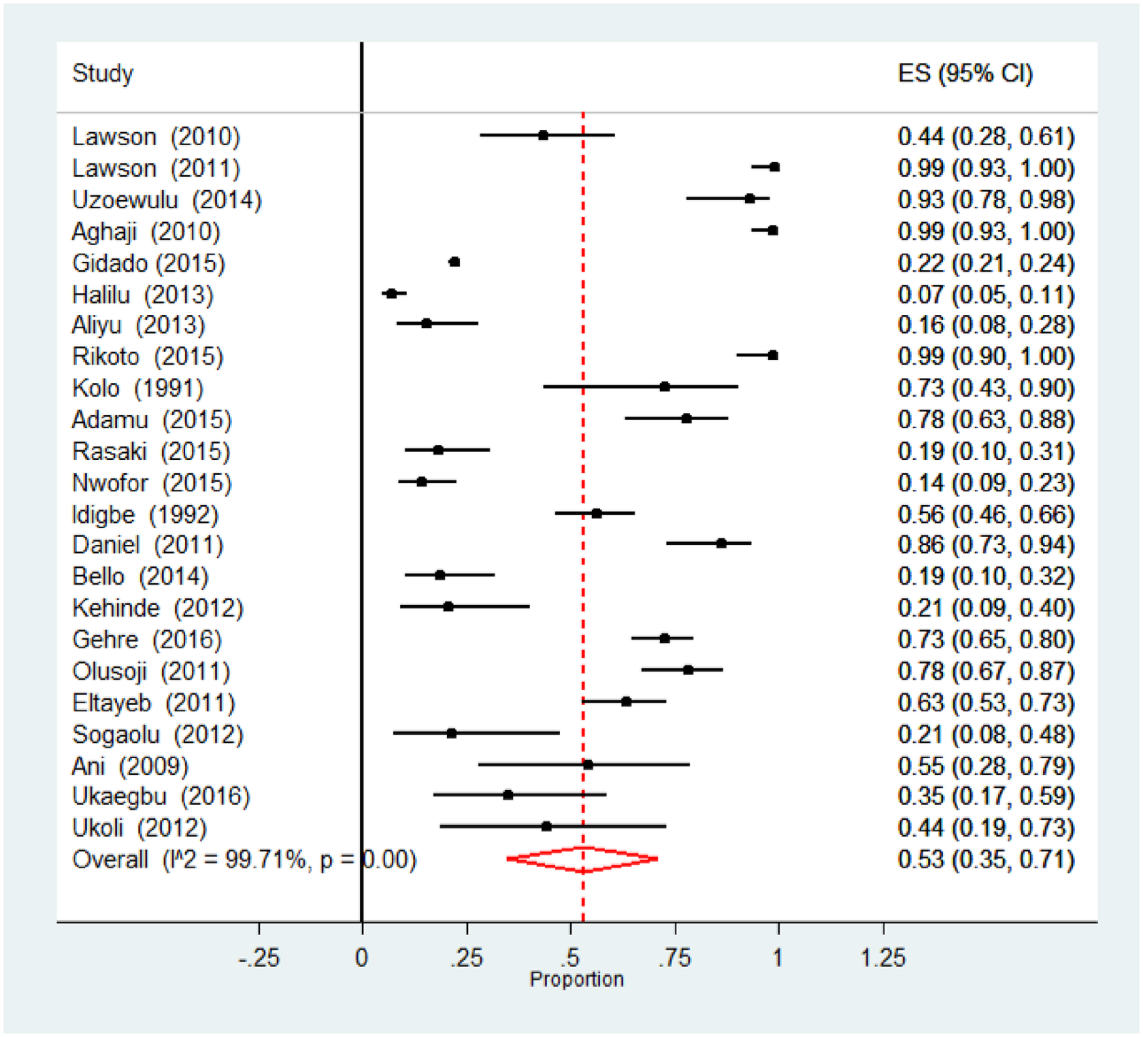
Forest plot of the meta-analysis on any drug resistance in previously-treated cases. [CI: confidence interval].

**Fig 6:**
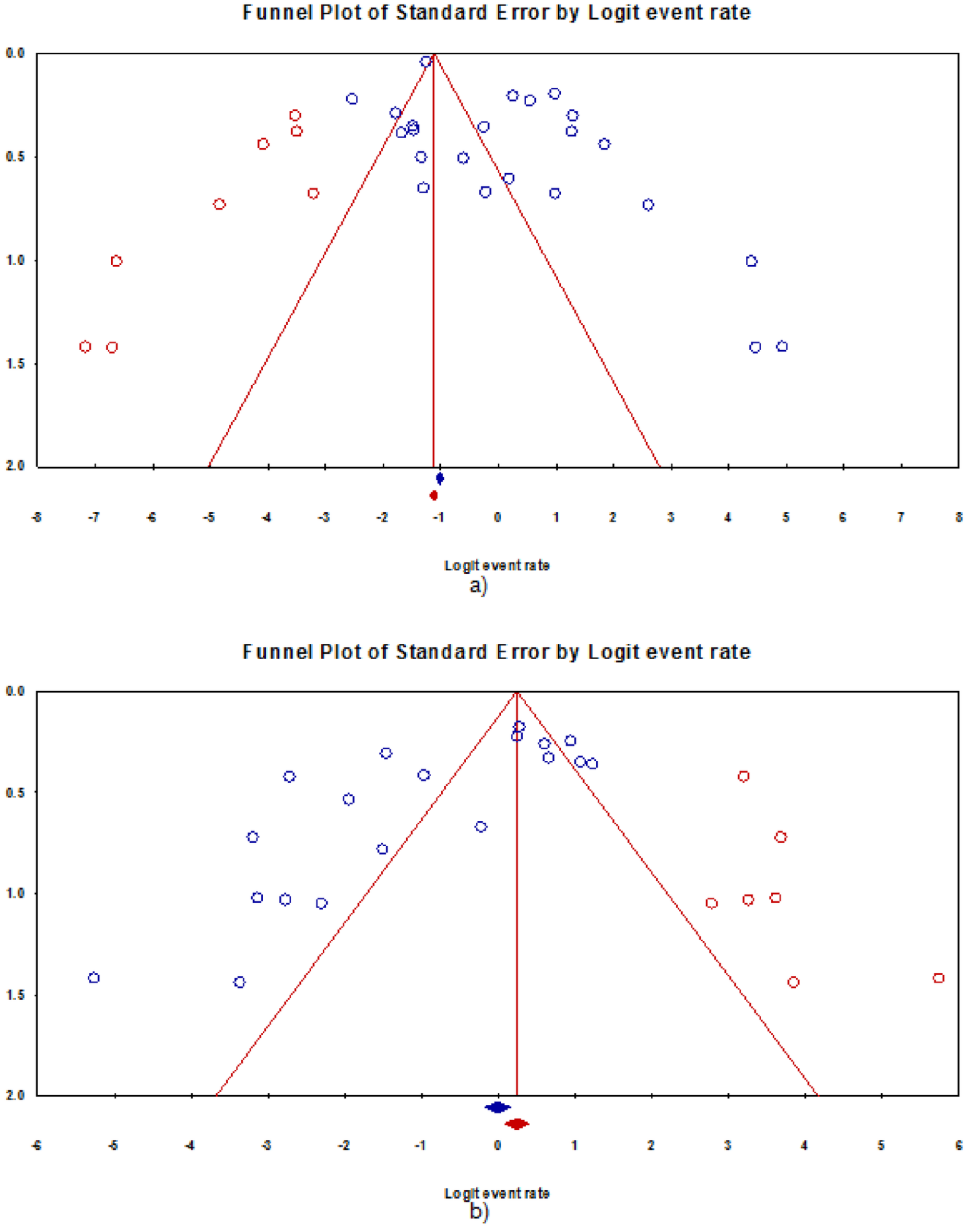
Funnel plot of the meta-analysis on prevalence of: a) any drug resistance, b) multidrug resistance among previously-treated TB patients, Nigeria.

The pooled analyses of the burden of DR-TB among previously-treated TB patients in Nigeria are as shown in Table 3. The rate of any resistance among previously-treated TB patients was 53.0% (95% CI 35.0–71.0%; 1467/5020). Fig 5 shows the forest plot of the meta-analysis of any anti-tuberculosis resistance in previously-treated TB patients. There was no statistical evidence for publication bias (Begg rank correlation analysis P = 0.37); however analysis of the funnel plot Fig 6 suggest that there is publication bias (Duval and Tweedie’s trim and fill indicate that there might be eight missing studies to the left of the mean). There was significant heterogeneity between studies (p<0.001). In addition, there was a wide variation in the rate of any resistance among previously-treated TB cases between studies utilising phenotypic and genotypic methods of DST (62.0 vs 26.0%) respectively (χ^2^ = 11.8, P <0.001). Furthermore, pooled rates for northern region (36.0%) was significantly lower compared with 62.0% observed among previously-treated TB patients in Southern Nigeria (χ^2^ = 4.76, P = 0.03). Overall the rate of mono-resistance to anti-TB drugs among previously-treated TB patients was 17.0% (95% CI 11.0–23.0; 127/789). This did not substantially vary across region and DST categories, Table 3. The pooled prevalence of MDR-TB among previously treated TB patients was 32.0% (95%CI 20.0–44.0; 357/949); there was some variation in the rate according to DST method—occurring in 19.0% and 36.0% of patients diagnosed using genotypic and phenotypic methods, respectively; (χ^2^ = 1.71, P = 0.19). Some evidence of publication bias was observed (Begg rank correlation analysis P = 0.06), and analysis of the funnel plot, Fig 6 suggest that there might be some publication bias (Duval and Tweedie’s trim and fill indicate that there might be seven missing studies to the right of the mean). Also, the rate of MDR-TB among previously-treated TB patients in Nigeria was 26.0% in the northern and 40.0% in the southern region (χ^2^ = 1.02, P = 0.31). S2 Fig shows the forest plot of the meta-analysis of MDR-TB in previously-treated TB cases in Nigeria.

**Table 3:** Pooled event rates of drug resistant tuberculosis among previously-treated TB patients, Nigeria.

Variables	Sub-group	Pooled event rate % (95% CI)	n/N	No. of Studies	Heterogeneity I^2^ (P -value)
**Any drug resistance**	Total	53.0 (35.0–71.0)	1467/5020	23	99.5 (<0.001)
	Stratified by DST				
	Genotypic	26.0 (16.0–36.0)	907/4204	8	95.7 (0.001)
	Phenotypic	62.0 (44.0–80.0)	560/816	15	98.2 (0.001)
	Stratified by region^a^				
	North	36.0 (22.0–51.0)	167/666	11	94.7 (0.001)
	South	62.0 (44.0–80.0)	416/616	10	97.8 (0.001)
**Monodrug resistance**	Total	17.0 (11.0–23.0)	127/789	16	85.3% (0.001)
	Stratified by DST				
	Genotypic	13.0 (8.0–19.0)	19/133	4	0.0 (0.51)
	Phenotypic	18.0 (11.0–25.0)	108/656	12	88.5 (<0.001)
	Stratified by region^a^				
	North	19.0 (12.0–27.0)	53/303	8	63.9 (0.01)
	South	15.0 (0.07–0.23)	74/486	8	89.8 (<0.001)
**Multidrug resistance**	Total	32.0 (20.0–44.0)	357/949	19	97.9 (<0.001)
	Stratified by DST method				
	Genotypic	19.0 (1.0–39.0)	31/133	4	95.3 (<0.001)
	Phenotypic	36.0 (21.0–51.0)	326/816	15	98.3 (<0.001)
	Stratified by region^a^				
	North	26.0 (10.0–41.0)	79/312	9	95.0 (0.001)
	South	40.0 (17.0–63.0)	265/568	9	98.8 (0.001)

DST = drug susceptibility testing method; TB = tuberculosis;

^a^ = studies from multiple regions excluded

## Discussion

Our analyses showed that 32.0% of newly diagnosed cases and 53.0% of previously-treated TB patients from different settings in Nigeria were resistant to at least one anti-TB medication. Furthermore, we found that the burden of MDR-TB was high—occurring in 6.0% of new and 32.0% of previously-treated TB patients. In addition, we found that the pooled burden of DR-TB across new and previously-treated cases varied substantially across geographic regions of the country and the DST methods used.

In this study, we found that almost a third of newly-diagnosed TB patients were resistant to at least one first-line anti-TB drugs. This suggests that there is a high rate of primary drug resistance among treatment-naive individuals diagnosed for the first time with TB. The existence of high rates of DR-TB among treatment-naïve individuals may be a reflection of active transmission of DR-TB in the community from the infectious DR-TB patients who are not on treatment. The prevalence of any DR-TB observed in this study is similar to the recent comprehensive estimates from Iran, China and Ethiopia; but lower than rates reported from Burundi and Portugal [7–8, 51–53]. In addition, the 6.0% prevalence of MDR-TB among new patients observed in this study is above the current WHO estimates of 4.3% (3.2–5.4%) for Nigeria [1]. This suggests that the burden of MDR-TB among new cases may be grossly underestimated and greater programmatic strategies are needed to detect and treat them. Since 2016, the NTP has adopted an algorithm positioning Xpert MTB/RIF as the initial diagnostic test for all persons with signs and symptoms of pulmonary TB [1]. It is expected that as more cases of DR-TB cases are detected and treated there will be a reduction in community transmission of primary anti-TB drug resistance in the country.

Also, we found that over half of previously-treated TB patients in Nigeria were resistant to at least one first-line anti-TB drug, and overall 32.0% of this group of patients had MDR-TB. This indicates that there is a high level of acquired resistance to anti-TB medications in the country. The pooled prevalence of any drug resistance among previously-treated TB patients observed fell within the range in Ethiopia (11.1 to 72.9%) and was similar to the rate reported by a review in China (49.8%), but lower than the rate reported from Iran (65.6%) [8–9, 51]. This indicates that in low- and middle-income countries there may be high rates of acquired resistance to anti-TB medications. The most common determinant of the occurrence of this drug resistance is the failure of the appropriate treatment of TB patients. Programmatically this could be from improper prescription of anti-TB treatment regimens, inadequate drug supply, poor quality of drugs, high default and treatment failure rates [3–6, 54–56]. In addition, once selected, drug resistant strains of *M*. *tuberculosis* may be transmitted in the community. From our review, we cannot determine the drivers of drug resistant TB in Nigeria hence the need for further studies. In our meta-analysis, 32.0% of previously-treated cases had MDR-TB. The rate of MDR-TB observed among previously-treated cases was higher than the global average (20.5%) and the current WHO estimates for Nigeria (25%) (19–31%) [1]. This suggests that the NTP need to strengthen the management of drug susceptible TB in order to bridge the gaps identified; and conduct a nationwide survey of DR-TB in Nigeria to provide a more accurate estimate of the burden, to inform programmatic management of DR-TB.

In this study, stratified analyses were performed according to the geographic region and DST method used. Except two studies that collected data from multiple regions of Nigeria, most of the included studies were conducted either in the northern or southern region of Nigeria. Our analyses for both new and previously-treated TB patients indicate that there is a distinct pattern in the rates of DR-TB in Nigeria—overall the rates were lower in the northern compared to the southern part of the country. For example, among new patients, the rate of any resistance in the northern versus southern part of Nigeria was 21.0% and 36.0%, respectively. Similarly, among previously treated TB patients the rates of any resistance in the northern versus southern part of Nigeria was 36.0% and 62.0%, respectively. This north-south disparity was also observed in the rates of MDR-TB among new and previously-treated TB cases. Although the regional differences between the estimates for most of the sub-groups in the northern and southern region of Nigeria did not reach statistical significance (probably because of the small sample size in most of the studies), the observed differences may be because most early studies on DR-TB were conducted in southern Nigeria where the only TB national reference laboratory available then was located. At least one TB reference laboratory is now available per zone in the country; these findings suggest a need to strengthen the detection and treatment of DR-TB cases in all parts of the country. Also, we observed a variation in the rates of DR-TB according to DST technique with studies utilising genotypic methods detecting fewer rates of resistance compared with those that used phenotypic methods. This may be because current genotypic methods limit their anti-TB drug resistance screening to one or two anti-TB drugs only, and again most of the earlier studies included utilised phenotypic methods (23 phenotypic verses 11 genotypic).

Our study had several strengths. In total we identified 34 anti-TB drug resistance surveys, which allowed us to pool results from 8002 patients with TB who underwent DST for possible detection of resistance. There are some limitations, however. First, our analyses may not fully represent the prevalence of drug resistance in TB in Nigeria because the magnitude of drug resistance has not yet been fully investigated in some parts of the country particularly the north-eastern zone (1 study only) which has been facing security challenges from the unpredictable Boko Haram terrorist group in the last decade. Second, due to unavailability of data from the primary studies, the potential effect of age, sex, ethnicity, socioeconomic status and life style of the patients on DR-TB prevalence could not be analysed. Third, although Nigeria has a high burden of TB-HIV co-infection [1, 57]; due to unavailability of data, we did not evaluate for the impact of HIV on DR-TB in Nigeria. Two recent reviews indicate that HIV is not a driver of DR-TB in Sub-Saharan Africa [58–59]. Fourth, due to inadequate number of studies, we are unable to conduct a meta-regression analysis to evaluate for statistical differences in prevalence of drug-resistant TB in Nigeria between and within region and DST methods. Finally, although our analyses indicated some evidence for its existence, potential publication bias could not be completely excluded.

In conclusion, our systematic review and meta- analysis showed high levels of MDR-TB more than the WHO estimates for Nigeria. This study has important policy implications. Due to the high rate of anti-TB drug resistance observed, this study affirms the Nigeria National Tuberculosis Programme’s decision to switch to molecular DST screening test as the initial diagnostic test for all people with signs and symptoms of pulmonary TB. Furthermore, it indicates the need to strengthen strategies for the detection and programmatic management of drug-susceptible and drug-resistant TB in Nigeria, as well as sustained monitoring of anti-TB drug resistance in the country. It is our recommendation that a national anti-TB drug resistance survey be carried out in all geopolitical regions of Nigeria to determine the actual prevalence of DR-TB using the existing available genotypic DST methods. This survey should also assess the potential effects of social and economic determinants (e.g., age, sex, place of residence, socio-economic status, literacy, family income) on the prevalence of DR-TB in Nigeria.

## Supporting information

S1 Table Search strategy used for one of the databases. (DOCX)

S2 Table Quality assessment of the included studies. (DOCX)

S3 Table Papers included in the systematic review. (DOCX)

S4 Table Papers excluded in the systematic review (with reasons). (DOCX)

S5 Table PRISMA checklist of the systematic review. (DOC)

S1 Fig Forest plot of the meta-analysis of MDR-TB in new TB cases in Nigeria. (TIF)

S2 Fig Forest plot of the meta-analysis of MDR-TB in previously-treated TB cases in Nigeria. (TIF)
