# Supplementary material for: Prevalence of drug-resistant tuberculosis in Nigeria: A systematic review and meta-analysis
Source: PLoS One. 2017 Jul 13;12(7):e0180996. doi: 10.1371/journal.pone.0180996 (PMC5509256; doi:10.1371/journal.pone.0180996)
Supplement: S1 Table — (DOCX) [file pone.0180996.s001.docx]

**S1 Table. Search strategy used for one of the databases**

| Medline/Pubmed | | | |  |
| --- | --- | --- | --- | --- |
| Group | | Search terms |  |  |
|  |  | **MeSH** (subterms in MeSH) | *Non MeSH* | Citations |
| #1 |  | Tuberculosis  Mycobacterium tuberculosis |  |  |
| #2 |  | Drug resistance  Drug susceptibility  Rifampicin  Isoniazid  Ethambutol  Pyrazinamide  Antitubercular agents | Anti-tuberculosis resistance  Rifampicin-resistant tuberculosis  Isoniazid-resistant tuberculosis  Ethambutol-resistant tuberculosis  Pyrazinamide-resistant tuberculosis  Drug-resistant tuberculosis  Multidrug-resistant tuberculosis |  |
| #3 |  | Nigeria |  |  |
| #1 AND #2 AND #3 | | | | 223 |
