# Supplementary material for: Prevalence of drug-resistant tuberculosis in Nigeria: A systematic review and meta-analysis
Source: PLoS One. 2017 Jul 13;12(7):e0180996. doi: 10.1371/journal.pone.0180996 (PMC5509256; doi:10.1371/journal.pone.0180996)
Supplement: S2 Table — (DOCX) [file pone.0180996.s002.docx]

**Table S1: Quality assessment of the included studies**

| Author and year | 1) Target population | 2)Recruitment | 3) Sample size adequacy | 4) Subject & setting | 5) Data analysis / response rate | 6) Standard measurement | 7) Reliable measurement | 8) Statistical analysis | 9) Confounder accounted for | 10) Sub-groups  identified | Quality items met (n/10) |
| --- | --- | --- | --- | --- | --- | --- | --- | --- | --- | --- | --- |
| Dosunmu 2008 | Yes | Yes | Yes | Yes | No | Yes | Yes | No | No | No | 6 |
| Lawson 2010 | No | Yes | No | Yes | Yes | Yes | Yes | Yes | No | Yes | 7 |
| Lawson 2011 | Yes | Yes | Yes | Yes | Yes | Yes | Yes | Yes | No | Yes | 9 |
| Uzoewulu 2014 | Yes | Yes | Yes | Yes | Yes | Yes | Yes | No | No | Yes | 8 |
| Nwachukwu 2016 | Yes | Yes | Yes | Yes | Yes | Yes | Yes | No | No | NA | 8 |
| Pokam 2013 | No | Yes | No | Yes | Yes | Yes | Yes | No | No | Yes | 6 |
| Otu 2013 | No | Yes | No | Yes | Yes | Yes | Yes | Yes | No | Yes | 7 |
| Aghaji 2010 | Yes | Yes | Yes | Yes | Yes | Yes | Yes | Yes | No | Yes | 9 |
| Gidado 2015 | Yes | Yes | Yes | Yes | Yes | Yes | Yes | No | No | No | 7 |
| Halilu 2013 | Yes | Yes | Yes | Yes | No | Yes | Yes | No | No | No | 6 |
| Aliyu 2013 | Yes | Yes | Yes | Yes | Yes | Yes | Yes | Yes | No | Yes | 9 |
| Fawcett 1975 | Yes | Yes | No | Yes | No | Yes | Yes | No | No | No | 5 |
| Rikoto 2015 | Yes | Yes | No | Yes | Yes | Yes | Yes | Yes | No | Yes | 8 |
| Kolo 1991 | Yes | Yes | No | Yes | Yes | Yes | Yes | No | No | Yes | 7 |
| Adamu 2015 | Yes | Yes | Yes | Yes | Yes | Yes | Yes | No | No | Yes | 8 |
| Rasaki 2015 | Yes | Yes | No | Yes | Yes | Yes | Yes | No | No | No | 6 |
| Nwofor 2015 | Yes | No | No | Yes | Yes | Yes | Yes | No | No | No | 5 |
| Idigbe 1992 | Yes | Yes | No | Yes | Yes | Yes | Yes | No | No | No | 6 |
| Egbe 2016 | Yes | Yes | No | Yes | Yes | Yes | Yes | No | No | No | 6 |
| Daniel 2011 | Yes | Yes | No | Yes | No | Yes | Yes | No | No | Yes | 6 |
| Oluwaseun 2013 | Yes | Yes | No | Yes | No | Yes | Yes | No | No | No | 5 |
| Okodua 2012 | Yes | Yes | No | Yes | Yes | Yes | Yes | No | No | No | 6 |
| Bello 2014 | Yes | Yes | No | Yes | Yes | Yes | Yes | No | No | No | 6 |
| Kehinde 2012 | Yes | Yes | No | Yes | Yes | Yes | Yes | No | No | No | 6 |
| Kehinde 2013 | Yes | Yes | No | Yes | No | Yes | Yes | No | No | No | 5 |
| Kehinde 2007 | Yes | Yes | Yes | Yes | Yes | Yes | Yes | No | No | No | 7 |
| Gehre 2016 | Yes | Yes | Yes | Yes | Yes | Yes | Yes | Yes | Yes | Yes | 10 |
| Olusoji 2011 | Yes | Yes | Yes | Yes | Yes | Yes | Yes | Yes | Yes | Yes | 10 |
| Eltayeb 2011 | Yes | Yes | Yes | Yes | Yes | Yes | Yes | No | No | NA | 7 |
| Sogaolu 2012 | Yes | Yes | Yes | Yes | Yes | Yes | Yes | No | No | Yes | 8 |
| Ani 2009 | Yes | Yes | No | Yes | Yes | Yes | Yes | No | No | Yes | 7 |
| Mawak 2006 | Yes | Yes | No | Yes | Yes | Yes | Yes | No | No | NA | 7 |
| Ukaegbu 2016 | Yes | Yes | No | Yes | Yes | Yes | Yes | No | No | Yes | 7 |
| Ukoli 2012 | Yes | Yes | No | Yes | No | Yes | Yes | No | No | No | 5 |

NA = not applicable, Unclear, High quality: 19, Moderate quality 15
