# Supplementary material for: Prevalence of drug-resistant tuberculosis in Nigeria: A systematic review and meta-analysis
Source: PLoS One. 2017 Jul 13;12(7):e0180996. doi: 10.1371/journal.pone.0180996 (PMC5509256; doi:10.1371/journal.pone.0180996)
Supplement: S3 Table — (DOCX) [file pone.0180996.s003.docx]

**Table S2: Papers cited (Included in the Systematic Review)**

1. Dosunmu E A, Osagie K, Shuaib A, Lawson L. Multidrug-resistant tuberculosis at the National Hospital, Abuja, Nigeria. Afr J Respir Med 2008; 4: 22-23.
2. Lawson L, Habib A G, Okobi M I, et al. Pilot study on multidrug resistant tuberculosis in Nigeria. Ann Afr Med 2010; 9:184-187.
3. Lawson L, Yassin M A, Abdurrahman S T, et al. Resistance to first-line tuberculosis drugs in three cities of Nigeria. Trop Med Int Health 2011; 16:974-980.
4. Uzoewulu N G, Ibeh I N, Lawson L, et al. Drug resistant Mycobacterium tuberculosis in tertiary hospital South East, Nigeria. J Med Microb Diagn 2014; 3:2
5. Nwachukwu N O, Onyeagba R A, Nwaugo V O, Ononiwu H A, Okafor D C. Diagnostic accuracy of Xpert MTB/RIF assay in diagnosis of pulmonary tuberculosis. J Infec Dis Treat. 2016; 2:1
6. Pokam B T, Asuquo A E, Abia-Bassey L N, et al. Multidrug resistance and demography of newly diagnosed tuberculosis patients in Cross River State, Nigeria. Int J Mycobacteriol 2013; 2:89-93.
7. Otu A, Umoh V, Habib A, Ameh S, Lawson L, Ansa V. Drug resistance among pulmonary tuberculosis patients in Calabar, Nigeria. Pulm Med 2013; 2013:235190.
8. Aghaji M N, Nwakoby B A N. Drug-resistance in chronic tuberculosis cases in Southern Nigeria. Niger J Clin Pract 2010;13:58-63
9. Mustapha G, Jumoke O, Nwadike P, et al. Assessment of Gene-xpert MTB RIF program implementation and the challenges for enhanced tuberculosis diagnosis in Nigeria. SAARC J Tuberc Lung Dis HIV/AIDS 2015 XII(2): 1 – 7
10. Halilu T B, Bala Z, Sado F, Yerima B I. Multi-drug resistance tuberculosis (MDR-TB) survey in North East Nigeria. J Pharm Cosmet Sci 2013; 1:45-52
11. Aliyu G, El-Kamary S S, Abimiku A, et al. Mycobacterial etiology of pulmonary tuberculosis and association with HIV infection and multidrug resistance in northern Nigeria. Tuberc Res Treat 2013;2013:650561.
12. Fawcett I W, Watkins B J. Initial resistance of Mycobacterium tuberculosis in Northern Nigeria. Tubercle 1975;57:71-73
13. Rikoto J A. Pattern of first-line anti-tuberculosis drug resistance and associated factors in patients attending national tuberculosis and leprosy training centre and referral hospital Zaria. 2015 PhD thesis: Zaria, Nigeria: Ahmadu Bello University.
14. Kolo I. Bacteriological and drug sensitivity studies on Mycobacteria isolated from tuberculosis patients and their close contacts in ABUTH, Zaria, Nigeria. 1991; PhD Thesis, Zaria, Nigeria.
15. Adamu A U, Hafiz T R. Multi-drug resistant tuberculosis pattern in Kano metropolis, Nigeria. J Am Sci 2015;11:293-296
16. Rasaki S O, Ajibola A A, Musa S A, et al. Rifampicin resistant tuberculosis in a secondary health institution in Nigeria, West Africa. J Infect Dis Ther 2014;2:3
17. Nwofor A C, Nyamngee A, Nwabuisi C, et al. Performance of genotype MTBDRplus in the detection of resistance to rifampicin and isoniazid among clinical mycobacteria isolates in Ilorin, Nigeria. Curr HIV Res 2015; 13: 308-314
18. Idigbe E O, Duque J P, John E K, Annam O. Resistance to antituberculosis drugs in treated patients in Lagos, Nigeria. J Trop Med Hyg 1992;95:18691.
19. Egbe K, Ike AC, Aleruchi C. Prevalence of tuberculosis and rifampicin resistance among patients seeking medical care in Nasarawa State, north central Nigeria. Sci J Public Health 2016;4:214-218
20. Daniel O, Osman E, Bakare R, et al. Ofloxacin resistance among Mycobacterium tuberculosis isolates in two states of south-west Nigeria. Afr J Respir Med 2011; 6:18-20
21. Oluwaseun E, Akinniyi A P, Afolabi O. Primary multi-drug resistant tuberculosis among HIV seropositive and seronegative patients in Abeokuta, southwestern Nigeria. Am J Res Comm 2013;1:224 – 237
22. Okodua M, Ihongbe J, Esumeh F. Pulmonary tuberculosis and resistance pattern to first line antituberculosis drugs in a city of western Nigeria. Int J Basic Appl Innov Res 2012;1: 48 – 56
23. Bello L A, Shittu M O, Shittu B T, Oluremi A S, Akinnuroju O N, Adekola S A. Rifampicin-monoresistant Mycobacterium tuberculosis among the patients visiting chest clinic, state specialist hospital, Akure, Nigeria. Int J Res Med Sci 2014;2:1134-1137
24. Kehinde A O, Adetoye A E. Diagnosis of pulmonary tuberculosis using genotype MTBDRPLUS assay in three local government primary health centres of Osun State, Nigeria- a pilot study. J Med Microb Diagn 2012;S3:001.
25. Kehinde A O, Adebiyi E O. Molecular diagnosis of MDR-TB using GenoType MTBDRplus 96 assay in Ibadan, Nigeria. Niger J Physiol Sci 2013; 28:187–191
26. Kehinde AO, Obaseki FA, Ishola OC, Ibrahim KD. Multidrug resistance to Mycobacterium tuberculosis in a tertiary hospital J Natl Med Assoc 2007;99:1185-9
27. Gehre F, Otu J, Kendall L, et al. The emerging threat of pre-extensively drug-resistant tuberculosis in West Africa: preparing for large-scale tuberculosis research and drug resistance surveillance. BMC Med 2016;14:160.
28. Olusoji D, Eltayeb O. Prevalence and risk factors associated with drug resistant TB in Southwest, Nigeria. Asian Pac J Trop Med 2011; 4:148-151.
29. Eltayeb O, Daniel O, Ogiri S, et al. Resistance of Mycobacterium tuberculosis to first and second line anti tuberculosis drugs in South West, Nigeria. J Pulmon Resp Med S6:001.
30. Sogaolu O M, Ige O M, Lawson L, Akinyemi J, Lawa O. Pattern of resistance to first line antituberculosis drugs in Ibadan, Nigeria - preliminary observations. Am J Respir Crit Care Med 2012;185:A3257
31. Ani A E, Idoko J, Dalyop Y B, Pitmang S L. Drug resistance profile of Mycobacterium tuberculosis isolates from pulmonary tuberculosis patients in Jos, Nigeria. Trans R Soc Trop Med Hyg 2009;103:67-71.
32. Mawak J D, Gomwalk N E, Bello C S S, Kandakai-Olukemi Y T. Drug susceptibility pattern of mycobacterium tuberculosis among, pulmonary tuberculosis patients in Jos, Nigeria. Nig J Exp Appl Biol 2006; 7: 128-133
33. Ukaegbu C O, Ani A, Nnachi A U. Molecular detection of mycobacterium tuberculosis complex by genotype®MTBDRPlus from patients attending Bingham University Teaching Hospital, Jos, Nigeria. Br J Pharm Res 2016; 9: 1 – 11
34. Ukoli C O, Akanbi M O, Adiukwu C V, Amusa G A, Akanbi F O. Diagnosing tuberculosis in resource limited settings: experience from a referral TB clinic in North Central Nigeria. Jos J Med 2012;6:26-27
