# Supplementary material for: Prevalence of drug-resistant tuberculosis in Nigeria: A systematic review and meta-analysis
Source: PLoS One. 2017 Jul 13;12(7):e0180996. doi: 10.1371/journal.pone.0180996 (PMC5509256; doi:10.1371/journal.pone.0180996)
Supplement: S4 Table — (DOCX) [file pone.0180996.s004.docx]

**Table S3: Papers Excluded in the Systematic review (with reasons)**

1. Okorie O, John A, Gidado M, et al. The Prevalence of Drug-Resistant Tuberculosis among People Living with HIV (PLHIV) in Abia State. Adv Infect Dis 2016; 6: 63-69. [**Insufficient/No required data**]
2. Nwadioha S I, Nwokedi E O P, Ezema GC, et al. Drug resistant *Mycobacterium tuberculosis* in Benue, Nigeria. Br Microbiol Res J 2014; 4: 988-995 [**Insufficient/No required data**]
3. Mbaave T P, Igbudu T J, Egwuda L, Audu O, Jombo G T. Gene Xpert MTB/RIF sputum MTB detection and human immunodeficiency virus seroprevalence among patients with presumptive pulmonary tuberculosis at a new teaching hospital in north-central Nigeria. Int J Curr Microbiol App Sci 2016;5: 564-570 [**Insufficient/No required data**]
4. Ochang E A, Udoh U A, Emanghe U E, et al. Evaluation of rifampicin resistance and 81-bp rifampicin resistant determinant region of rpoB gene mutations of Mycobacterium tuberculosis detected with XpertMTB/Rif in Cross River State, Nigeria. Int J Mycobacteriol 2016;5 Suppl 1:S145-S146. [**Insufficient/No required data**]
5. Otu A, Umoh V, Habib A, Ansa V. Prevalence and clinical predictors of drug-resistant tuberculosis in three clinical settings in Calabar, Nigeria. Clin Respir J 2014;8:234-239. [**Duplicate publication**].
6. Gidado M, Onazi O, Obasanya O, et al. Assessing the effectiveness of Xpert MTB/RIF in the diagnoses of TB among HIV smear negative TB patients in Nigeria. J Health Sci 2014;2: 145-151. [**Duplicate publication**]
7. Felkel M, Exner R, SchleucherR, et al. Evaluation of mycobacterium tuberculosis drug susceptibility in clinical specimens from Nigeria using genotype MTBDRPLUS and MTBDRSL assays. Eur J Microbiol Immunol (Bp) 2013;3: 252–257 [**Insufficient/No required data**]
8. Tope A T, Thomas B T, Agu G, Abiodun A. Can Nigeria sustain the fight against drug resistant Mycobacterium tuberculosis? J Microbiol Res 2014; 4:72-77 [**Review Article**]
9. Daniel O, Osman E, Oladimeji O, Dairo OG. Pre-extensive drug resistant tuberculosis (Pre-XDR-TB) among MDR-TB patients in Nigeria. Glo Adv Res J Microbiol 2013;2:22-28. [**Insufficient/No required data**]
10. Daniel O, Osman E, Awe A, Ogiri S, Lawal W, Sobaloju S. Antituberculosis drug resistance pattern among newly diagnosed pulmonary tuberculosis patients in south west Nigeria. Ann Afr Med 2011;10:256-7. . [**Duplicate publication**]
11. Isara A R, Akpodiete A. Concerns about the knowledge and attitude of multi drug-resistant tuberculosis among health care workers and patients in Delta State, Nigeria. Niger J Clin Pract 2015;18:664-9. [**Insufficient/No required data**]
12. Njoku C H, Isezuo S A, Anas S. Multi-drug resistant tuberculosis: two case reports and review of literature. J Med Trop 2004; 6: 19-25. [**Case report**]
13. Ochang E A, Odubeyo O O, Onwuezobe I A, Collier D, Bode-Sojobi I, Odo M. Feasibility and cost analysis of programmatic implementation of Microscopic-Observation Drug Susceptibility (MODS) assay in Nigeria. Niger J Med 2016; 25: 226-233 [**Insufficient/No required data**]
14. Dinic L, Akande P, Idigbe E O, et al. Genetic determinants of drug-resistant tuberculosis among HIV-infected patients in Nigeria. J Clin Microbiol 2012;50: 2905-2909 [**Insufficient/No required data**]
15. Bieh K L, Weigel R, Smith H. Hospitalized care for MDR-TB in Port Harcourt, Nigeria: a qualitative study. BMC Infect Dis 2017;17:50. [**Insufficient/No required data**]
16. Akanbi M O, Ukoli C O. Are weak health systems a brewing ground for multi-drug-resistant tuberculosis? Afr J Respir Med 2008; 5: 21-22 [**Case report**]
17. Nwokeukwu H I, Okafor P N, Okorie O, Ukpabi K. Paediatric multidrug resistant tuberculosis with HIV co infection: a case report. Case Rep Med 2013;2013:756152 [**Case report**]
18. Kehinde A O. Molecular epidemiologic tools for diagnosis of tuberculosis: A review of literature and its’ applicability in Nigeria. Afr J Microbiol Res 2012; 6: 6693-6697 [**Review Article**]
19. Akingbade O. Perspectives on community tuberculosis care in Nigeria. Int J Trop Dis Health 2016;16:1–13 [**Editorial**]
20. Anakwue R C, Onyedum C C. Management of Adverse Effects of Antituberculosis Drugs. J Coll Med 2008;13: 116-122 [**Insufficient/No required data**]
