# Supplementary figures and images for: Prevalence of drug-resistant tuberculosis in Nigeria: A systematic review and meta-analysis
Source: PLoS One. 2017 Jul 13;12(7):e0180996. doi: 10.1371/journal.pone.0180996 (PMC5509256; doi:10.1371/journal.pone.0180996)

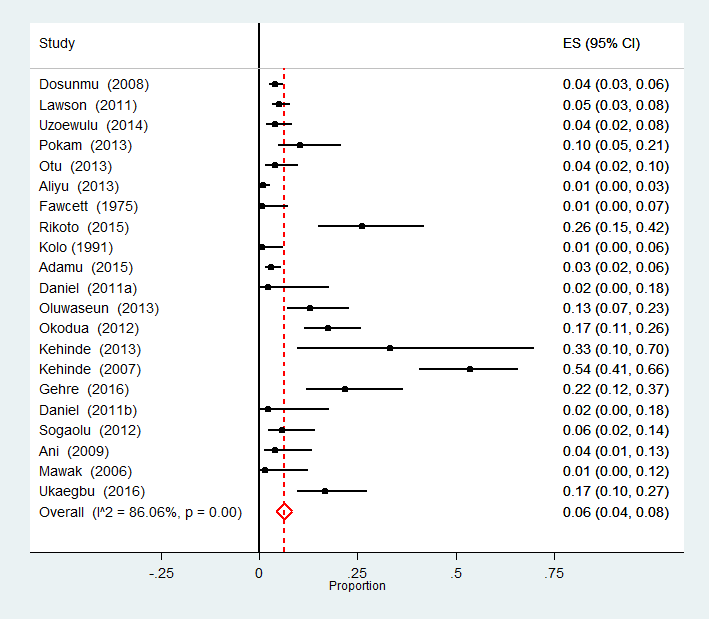

Supplement: S1 Fig — (TIF) [file pone.0180996.s006.tif]

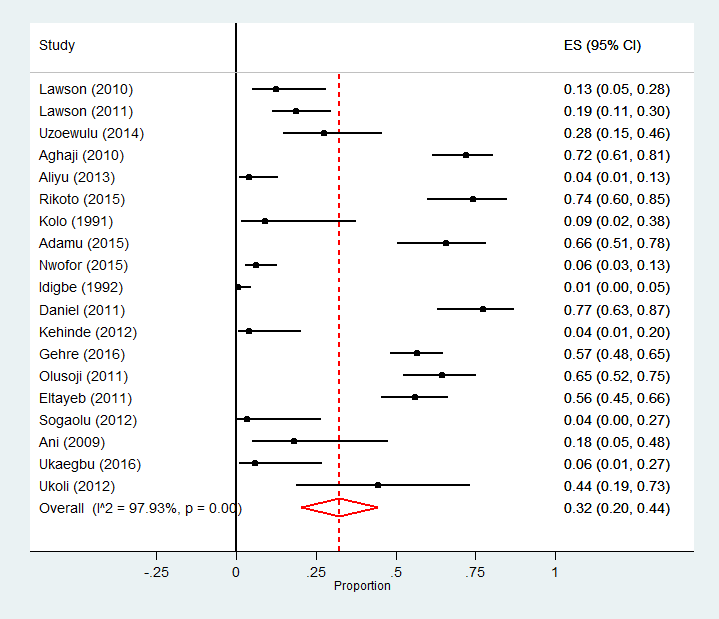

Supplement: S2 Fig — (TIF) [file pone.0180996.s007.tif]
